# Interferon lambda rs368234815 ΔG/ΔG is associated with higher CD4^+^:CD8^+^ T-cell ratio in treated HIV-1 infection

**DOI:** 10.1186/s12981-020-00269-0

**Published:** 2020-04-15

**Authors:** Inês T. Freitas, Willard Tinago, Hirofumi Sawa, Julie McAndrews, Brenda Doak, Charlotte Prior-Fuller, Gerard Sheehan, John S. Lambert, Eavan Muldoon, Aoife G. Cotter, William W. Hall, Patrick W. G. Mallon, Michael J. Carr

**Affiliations:** 1 Centre for Experimental Pathogen Host Research, School of Medicine, University College Dublin, Dublin 4, Ireland; 2 Global Station for Zoonosis Control, Global Institution for Collaborative Research and Education (GI-CoRE), Hokkaido University, N20, W10, Kita-ku, Sapporo, 001-0020 Japan; 3 Global Virus Network (GVN), 801 W. Baltimore St., Baltimore, MD 21201 USA; 4 Department of Immunology, Mater Misericordiae University Hospital, Dublin 7, Ireland; 5 National Virus Reference Laboratory, School of Medicine, University College Dublin, Dublin 4, Ireland; 6 Department of Infectious Diseases, St Vincent’s University Hospital, Dublin 4, Ireland; 7 Department of Infectious Disease, Mater Misericordiae University Hospital, Dublin 7, Ireland

**Keywords:** HIV, Interferon lambda, T-cells, CD4^+^, CD8^+^

## Abstract

**Background:**

The objectives of this study were to investigate the relationships between polymorphisms at the interferon lambda (*IFNL*) locus and CD4^+^:CD8^+^ ratio normalisation in people living with HIV (PLWH) on effective antiretroviral therapy (ART); and to examine whether these polymorphisms influence the composition of T lymphocyte compartments in long-term treated HIV-1 infection.

**Methods:**

A cross-sectional study in PLWH enrolled into the Mater Immunology study. We performed *IFNL* genotyping on stored samples and evaluated the association of *IFNL* single-nucleotide polymorphisms (rs368234815 and rs12979860) with CD4^+^:CD8^+^ ratio normalization (> 1) and expanded CD4^+^ and CD8^+^ T-cell subsets; CD45RO^+^CD62L^+^ (central-memory), CD45RO^+^ CD62L^−^(effector-memory) and CD45RO^−^CD62L^+^ (naïve), using logistic and linear regression models, respectively.

**Results:**

190 ambulatory PLWH recruited to the main study, 143 were included in the analysis (38 had no stored DNA and 9 no T-lymphocyte subpopulation). Of 143 included, the median age (IQR) was 45(39–48) years, 64% were male and 66% were of Caucasian ethnicity. Heterosexual-contact (36%), injecting drug-use (33%) and men who have sex with men (24%) were the most presented HIV-transmission risk groups. The majority of subjects (90.2%) were on ART with 79% of the cohort having an undetectable HIV-RNA (< 40 copies/ml) and the time since ART initiation was 7.5 (3.7–10.4) year. rs368234815 and rs12979860 displayed similar allelic frequencies, with minor alleles ΔG and T representing 39% and 42%, respectively, of circulating alleles. rs368234815 ΔG/ΔG minor homozygotes were significantly associated with increased odds for attaining a normalised CD4^+^:CD8^+^ ratio compared to rs368234815 T/T major homozygotes in PLWH virologically suppressed on effective ART (OR = 3.11; 95% CI [1.01:9.56]). rs368234815 ΔG/ΔG homozygosity was also significantly associated with lower levels of CD4^+^ effector memory T-cells (regression coefficient: − 7.1%, *p *= *0.04*) and CD8^+^ naïve T-cell subsets were significantly higher in HIV-1 mono-infected PLWH with rs368234815 ΔG/ΔG (regression coefficient: + 7.2%, *p *= *0.04*).

**Conclusions:**

In virally-suppressed, long-term ART-treated PLWH, rs368234815 ΔG/ΔG homozygotes were more likely to have attained normalisation of their CD4^+^:CD8^+^ ratio, displayed lower CD4^+^ effector memory and higher naive CD8^+^ T-cells. Further studies are needed to replicate our findings in other, larger and more diverse cohorts and to determine the impact of *IFNL* genetic-variation on CD4^+^:CD8^+^ ratio normalisation and clinical outcomes in PLWH.

## Introduction

With advances in antiretroviral therapy (ART), HIV-infection has become a chronic, manageable condition. For the majority of individuals, infection with HIV-1 results in inversion of the normal CD4^+^:CD8^+^ ratio and, despite effective ART, only about 30% of subjects on ART for more than 5 years achieve normalisation of their CD4^+^:CD8^+^ T-cell ratios to > 1 [1]. The clinical implications of persistently low CD4^+^:CD8^+^ T-cell ratio are increasingly recognized, with lower ratios associated with greater risk of non-AIDS morbidity and mortality [2, 3]. As a result, attention has turned to understanding factors that influence poorer immune responses to ART, including failure to normalise the CD4^+^:CD8^+^ T-cell ratio. Initiation of ART early after HIV acquisition or at higher CD4^+^ T-cell counts is associated with higher subsequent CD4^+^:CD8^+^ ratios [4–6], and ART containing the non-nucleoside reverse transcriptase inhibitor efavirenz [7] or integrase strand transfer inhibitors (InSTI) [8] also show a stronger association with T-cell ratio normalisation compared to other ART regimens

More recently, the role of the host immune system in determining CD4^+^:CD8^+^ T-cell ratio responses to ART have also been explored. Two cohort studies have demonstrated that subjects with higher naïve CD8^+^ T-cell populations had higher CD4^+^:CD8^+^ T-cell ratios on ART [9, 10], while higher CD4^+^:CD8^+^ T-cell ratios prior to infection or the presence of human leukocyte antigen (*HLA*)-A*74:01 in an African cohort [11] have also been reported to positively influence normalisation of CD4^+^:CD8^+^ T-cell ratios. Conversely, co-infections with other viruses such as cytomegalovirus (CMV) and hepatitis C virus (HCV) [4, 5, 12], or the CD4^+^ T-cell apoptosis-inducing phenotype of the HIV Env glycoprotein envelope proteins and immune activation [13], all have been reported to decrease the likelihood of CD4^+^:CD8^+^ T-cell ratio normalisation.

Host genetics can also influence immunological responses to viral infections. Several polymorphisms near the type III interferon lambda (*IFNL3*) locus, previously named interleukin *28B* (*IL28B*), are able to predict both spontaneous clearance of hepatitis C infection by the host as well as better responses to pegylated interferon-alpha/ribavirin (PEG/RBV) therapy for HCV infection [14–17]. Similarly, the rs12979860 C>T single nucleotide polymorphism (SNP) is associated with higher HCV RNA titers and improved response to PEG/RBV therapy in those carrying the CC major homozygous genotype [14, 18, 19]. The rs12979860 polymorphism displays high linkage disequilibrium with a dinucleotide/deletion variant (TT/∆G) rs368234815 (TT>ΔG) [20]. While the TT/TT genotype represents a frameshift that abrogates IFNL4 expression, presence of ∆G allows for expression of IFNL4 [20]. IFNLs play a key role in innate immunity and antimicrobial defense of mucosal tissues [21] and recent data shows an effect on adaptive immune responses by both induction of thymic stromal lymphopoietin (TSLP) and effects on migratory dendritic cells [22]. Presence of the minor allele rs368234815 (ΔG) has been associated with poorer immune responses to CMV infection and higher risk of CMV retinitis in a cohort with advanced HIV-1 infection (nadir CD4^+^ T-cell counts of < 100 cells /mm^3^) [23], while rs368234815 (ΔG/ΔG) homozygous individuals undergoing solid organ transplant from CMV seropositive donors had a significantly higher cumulative incidence of CMV reactivation [24].

Whether expression of IFNL4 in HIV-1 infection modifies immune responses, particularly in those PLWH on effective ART, remains unclear, with contrasting observations reported. The presence of rs12979860 polymorphisms did not influence HIV acquisition in cohorts of individuals at high risk for HIV [25, 26] and was not over-represented within a cohort of African-American subjects with HIV who were classified as elite controllers [27]. In contrast, injecting drug users with HCV infection carrying the major homozygous rs368234815 (T/T) alleles were less likely to acquire HIV, [28] while presence of the rs368234815 ΔG allele was associated with higher prevalence of AIDS-defining illnesses and lower CD4^+^ T-cell counts in a cohort of asymptomatic, ART-naïve PLWH [29].

While IFNLs have been shown to skew T lymphocyte responses, their impact in treated HIV, in particular the potential for IFNL4 expression to modulate the CD4^+^:CD8^+^ T-cell ratio in the context of treated HIV-1 infection has not been explored to date. The objectives of the present study were to: (i) investigate relationships between polymorphisms at the *IFNL* locus and CD4^+^:CD8^+^ ratio normalisation in PLWH on effective ART; and (ii) examine whether these polymorphisms influence the composition of T lymphocyte compartments in long-term treated HIV-1 infection.

## Methods

### Study cohort and blood collection

We conducted a cross-sectional analysis exploring IFNL polymorphisms within a single-centre, prospective cohort study of PLWH designed to assess associations between CD4^+^ and CD8^+^ T lymphocyte subsets and CD4^+^:CD8^+^ T-cell ratio normalisation [9]. Briefly, the prospective cohort enrolled consecutive adult (>18 years old) PLWH attending the Mater Misericordiae University Hospital (MMUH) infectious diseases outpatients services for routine HIV clinical care, in Dublin, Ireland.

Enrolled subjects provided ethylenediaminetetraacetic acid (EDTA) blood used to determine expanded CD4^+^ and CD8^+^ T-cell subsets by flow cytometry; CD45RO^-^CD62L^+^ (naïve), CD45RO^+^CD62L^+^ (central memory) and CD45RO^+^CD62L^−^ (effector memory) (Additional file 1: Figure S1).alongside routine T-cell subsets (absolute and percentage CD4^+^ and CD8^+^ counts) and samples for storage. Additional assessments included measurements of HIV-RNA and collection of demographic and treatment data. The cross-sectional analysis was conducted on data derived from study entry [9]. All enrolled subjects provided written, informed consent and the study was approved by the Mater Misericordiae University Hospital and Mater Private Hospital Institutional Review Board.

### IFNL genotyping

Genomic deoxyribonucleic acid (DNA) was extracted from stored buffy coat cell pellets on the automated Magnapure 96 platform (Roche). *IFNL* genotyping was performed employing TaqMan SNP genotyping allelic discrimination on the ABI 7500 Fast instrument (Applied Biosystems, Warrington, United Kingdom). Assay conditions and thermocycling parameters for allelic discrimination real-time polymerase chain reaction (PCR) were as previously described for rs12979860 and validated by Sanger sequencing [18] and the rs368234815 SNP genotyping assay was supplied by Applied Biosystems. All genotyping was undertaken with blinding to clinical variables. The oligonucleotide primers and hydrolysis probes were as follows: rs12979860: rs12979860_F 5′GCCTGTCGTGTACTGAACCA; rs12979860_R 5′ GCGCGGAGTGCAATTCAAC; C allele probe (rs12979860_VIC) 5′VIC-TGGTTCGCGCCTTC-MGBNFQ; T allele probe (rs12979860_FAM) 5′FAM-CTGGTTCACGCCTTC-MGBNFQ; rs368234815: rs368234815_F 5′ CTCCAGCGAGCGGTAGTG; rs368234815_R 5′GGGTCCTGTGCACGGT; TT allele probe (ss469415590IF_VI) 5′VIC-ATCGCAGAAGGCC-MGBNFQ; ΔG allele probe (ss469415590IF_FAM) 5′FAM- ATCGCAGCGGCCC-MGBNFQ.

### Statistical analysis

Subject characteristics were summarised using descriptive statistics: median and interquartile range (IQR) for continuous, non-normal variables and frequencies/percentages for categorical variables. Accordance of the genotype frequencies with Hardy–Weinberg equilibrium was confirmed using Chi square test for each SNP. Regression models for assessing associations were restricted to those PLWH virologically suppressed on effective ART (HIV-RNA < 40 copies/ml). The genotype of each SNP was scored using an additive model: 0, homozygous for a major allele, 1, heterozygous for a major allele, and 2, homozygous for a minor allele.

As both presence of HIV viraemia and hepatitis C co-infection may impact on relationships between immune markers and genetic polymorphisms of interest, we analysed three main groups; the total cohort with available DNA, a subset of the cohort who were on ART with an undetectable HIV-RNA and a smaller cohort on ART without hepatitis C co-infection. Logistic regression models were used to examine the association of CD4^+^:CD8^+^ T-cell ratio normalisation (CD4^+^:CD8^+^ T-cell ratio ≥ 1 versus CD4^+^:CD8^+^ T-cell ratio < 1) with each genetic polymorphism. Linear regression analysis was used to investigate the association of CD4^+^ and CD8^+^ T-cell subsets (naïve, central memory and effector memory T-cells) with each genetic polymorphism. All tests were two-sided and analyses were performed using Stata 13 (StataCorp, College Station, Texas).

## Results

### Characteristics of the study subjects

Of 190 ambulatory PLWH recruited to the main study previously described [9], 152 with stored DNA were included in the present study. Of these, 9 (5%) did not have T lymphocyte subpopulation data available and were excluded from further analysis. The demographic characteristics of the 143 remaining subjects broadly reflected the European HIV epidemic, with male and female genders (63.6% male), major HIV transmission groups (heterosexual, injecting drug-users (IDU), men who have sex with men (MSM), needle stick) as well as Caucasian (65.7%) and African (28%) ethnicities represented. The majority of subjects (90.2%) were on ART with 79% of the cohort having an undetectable HIV-RNA (< 40 copies/ml) at the time of sampling. The median (IQR) time since HIV diagnosis and ART initiation were 10 [6–14] years and 7.5 (3.7–10.4) years respectively.

Subjects were genotyped for polymorphisms within the *IFNL* locus. Of the two genotypes tested, rs368234815 and rs12979860 displayed similar allelic frequencies, with minor alleles ΔG and T representing 39% and 42%, respectively, of circulating alleles. The minor homozygotes rs368234815 ΔG/ΔG and rs12979860 T/T showed comparable frequencies of 20% and 17%, respectively. The rs12979860 genotype had a higher number of heterozygous C/T genotypes (51%), with C/C major homozygotes detected in 32% of the subjects. Conversely, rs368234815 T/T major homozygosity was present in 43% of the subjects, with the remaining 37% carrying both alleles. Allele distribution did not appreciably change between the main study population and the two sub-populations studied (Table 1). There were no gender differences in the allele distribution for both rs368234815 (p = 0.12) and rs12979860 (p = 0.21). Compared to Caucasian ethnicity, non-Caucasians had significantly higher proportion of the minor homozygotes rs368234815 ΔG/ΔG (10% vs 41%, p < 0.001) and rs12979860 T/T (9% vs 33%, p < 0.001).

**Table 1:** Demographic characteristics of population under study

Characteristic	All (N = 143)	On ART with viral suppression (n = 111)	On ART, suppressed without HCV (n = 71)
Age (years, median (IQR)	45 (39–48)	44.0 (38.9–49.1)	42.8 (36.7–49.0)
Gender n (%)			
Female	52 (36.4%)	39 (35.1%)	27 (38.0%)
Male	91 (63.6%)	72 (64.9%)	44 (62.0%)
Ethnicity n (%)			
Caucasian	94 (65.7%)	76 (68.5%)	36 (50.7%)
African	40 (28%)	31 (27.9%)	31 (43.7%)
Other	9 (6.3%)	4 (3.6%)	4 (5.6%)
HIV-transmission risk group n (%)			
Heterosexual	52 (36.4%)	43 (38.8%)	41 (57.8%)
IDU	47 (32.9%)	38 (34.2%)	4 (5.6%)
MSM	35 (24.4%)	27 (24.3%)	24 (33.8%)
Needle stick/unknown	9 (6.3%)	3 (2.7%)	2 (2.8%)
Hepatitis C Ab: positive n (%)	50 (35.0%)	40 (36.0%)	
Hepatitis B sAg: positive n (%)	4 (2.8%)	4 (3.6%)	
Time since ART initiation (years, median (IQR))	7.5 (3.7–10.4)	7.8 (4.4–10.8)	8.6 (4.9–10.7)
HIV-RNA (copies/ml) n (%)			
≤ 40	113 (79%)		
ART exposure n (%)			
Experienced	129 (90.2%)		
ART regimes n (%)			
PI-based ART	72 (55.8%)	59 (53.2%)	
NNRTI based ART	52 (40.3%)	47 (42.3%)	
INSTI based ART	5 (3.9%)	5 (4.5%)	
rs12979860 n (%)			
C/C	46 (32.2%)	39 (35.1%)	22 (31.0%)
C/T	73 (51.0%)	57 (51.4%)	38 (53.5%)
T/T	24 (16.8%)	15 (13.5%)	11 (15.5%)
rs368234815 n (%)			
T/T	61 (42.6%)	48 (43.2%)	28 (39.5%)
T/ΔG	53 (37.1%)	43 (38.8%)	26 (36.6%)
ΔG/ΔG	29 (20.3%)	20 (18.0%)	17 (23.9%)

Data are n (%) unless otherwise stated

*HCV* hepatitis C, *IQR* interquartile range, *IDU* injecting drug use, *MSM* men who have sex with men, *ART* antiretroviral therapy, *SNP* single-nucleotide polymorphisms, *Ab-antibody* sAg-surface antigen, *INSTI* integrase inhibitor, *PI* proteases inhibitor, *NNRTI* non-nucleoside reverse transcriptase inhibitor NNRTI, PI, INSTI

### Relationship between *IFNL* locus polymorphism and CD4^+^: CD8^+^ T-cell ratio normalisation

In the study cohort, 33/143 (23%) PLWH had CD4^+^:CD8^+^ T-cell ratio ≥ 1. Compared to those with a non-normalised CD4^+^:CD8^+^ T-cell ratio, subjects who had a normalised T-cell ratio were more likely (74.6% versus 93.6%, *p *= 0.02) to have an undetectable HIV-RNA (< 40 copies/ml; Table 1). Consequently, all further analyses were restricted to PLWH with virological suppression on ART (n = 111). Demographic characteristics of this subpopulation did not notably differ from the main study cohort (Table 1).

Within the virologically suppressed population, subjects homozygous for rs368234815 (ΔG/ΔG) had significantly increased odds of T-cell ratio normalisation (odds ratio (OR) = 3.11, 95% CI [1.01; 9.56]), when compared to the major homozygote (T/T; Table 2). No significant association was observed between T-cell ratio normalisation and rs12979860 polymorphisms. The association between rs368234815 (ΔG/ΔG) and CD4^+^:CD8^+^ T-cell ratio normalization was slightly abrogated after correction for age, gender and treatment duration (OR = 2.80, 95% CI [0.82; 9.54]).

**Table 2:** Logistic regression for the associations between normalisation of the CD4^+^:CD8^+^ ratio and IFNL genotypes in PLWH on ART with viral suppression

SNP	Normalised (n = 31)	Not Normalised (n = 80)	Unadjusted OR (95% CI)	Adjusted OR (95% CI)^a^
rs12979860				
C/C	9	30	1 (referent)	1 (referent)
C/T	16	41	1.30 (0.51; 3.34)	1.31 (0.48; 3.55)
T/T	6	9	2.22 (0.62; 7.94)	2.28 (0.60; 8.69)
rs368234815				
T/T	10	38	1 (referent)	1 (referent)
T/ΔG	12	31	1.47 (0.56; 3.89)	1.69 (0.62; 4.60)
ΔG/ΔG	9	11	3.11 (1.01; 9.56)	2.80 (0.82; 9.54)

^a^Adjusted for age, gender, treatment duration and duration of HIV-infection

### Relationship between *IFNL* locus polymorphism and CD4^+^ and CD8^+^ T-cell subsets

In an exploratory analysis within the treated, virally suppressed population (n = 111), rs368234815 ΔG/ΔG homozygosity was also associated with significantly lower proportions of CD4^+^ effector memory T-cells (regression coefficient: − 7.1% (95% CI − 13.83; − 0.34) compared to the T/T genotype (Table 3). In a subpopulation of treated, virally suppressed Caucasian (n = 76), rs368234815 ΔG/ΔG homozygosity was associated with higher proportion of CD4^+^ and CD8 + central memory T-cells (regression coefficient: + 11.6% (95% CI 2.62; 20.50) and + 13.8 (95% CI 5.67; 21.97) respectively) compared to the T/T genotype. There was no association between the CD4 + and CD8 + T-cell subset in non-Caucasians.

**Table 3:** Linear regression for the relationships between T-cell subsets and IFNL genotypes in PLWH on ART with viral suppression (N = 111)

SNP	% CD4^+^ naïve			% CD4^+^ central memory			% CD4^+^ effector memory		
	Effect	95% CI	*p* value	Effect	95% CI	*p* value	Effect	95% CI	*p* value
rs12979860									
C/T	3.55	− 2.16; 9.25	0.22	− 1.59	− 6.00; 2.83	0.48	− 2.97	− 83.6; 2.42	0.28
T/T	2.00	− 6.27; 10.26	0.63	2.73	− 3.68; 9.13	0.40	− 3.58	− 11.40; 4.23	0.37
rs368234815									
T/ΔG	1.05	− 4.71; 6.81	0.72	0.62	− 3.78; 5.03	0.78	− 1.72	− 7.06; 3.62	0.53
ΔG/ΔG	2.51	− 4.76; 9.78	0.50	5.36	− 0.20; 10.92	0.06	− 7.08	− 13.83; − 0.34	0.04
rs12979860									
C/T	1.44	− 3.03; 6.19	0.55	2.19	− 1.91; 9.28	0.29	− 4.40	− 10.00; 1.19	0.12
T/T	2.55	− 4.33; 9.43	0.46	0.70	− 5.23; 6.64	0.82	− 1.63	− 9.73; 6.48	0.69
rs368234815									
T/ΔG	0.05	− 4.71; 4.80	0.98	3.10	− 0.99; 7.19	0.14	− 1.37	− 7.03; 4.29	0.63
ΔG/ΔG	3.03	− 2.98; 9.03	0.32	2.48	− 2.68; 7.64	0.34	− 3.42	− 10.57; 3.73	0.35

C/C is the reference group for rs12979860 and T/T is the reference group for rs368234815

Since SNPs in this *IFNL3/4* region have been extensively related to HCV infection outcomes, including treatment-induced and spontaneous viral clearance, associations between CD4^+^ and CD8^+^ T-cell sub-populations with *IFNL* SNPs were further investigated in a subgroup of virally suppressed PLWH without HCV coinfection (n = 71). Within this sub-population, rs368234815 ΔG/ΔG was associated with significantly higher proportions of naïve CD8^+^ T-cells (regression coefficient: + 7.2% (95% CI 0.28; 14.06) and lower proportions of effector memory CD8^+^ T-cells (− 7.6% (95% CI − 16.29; 1.05), albeit of marginal statistical significance, compared to the T/T genotype (Table 4).

**Table 4:** Linear regression for the relationships between T-cell subsets and IFNL genotypes in mono-infected PLWH on ART with viral suppression

SNP	% CD4^+^ naïve			% CD4^+^ central memory			% CD4^+^ effector memory		
	Effect	95% CI	*p* value	Effect	95% CI	*p* value	Effect	95% CI	*p* value
rs12979860									
C/T	3.97	− 3.84; 11.78	0.31	− 2.40	− 8.00; 3.20	0.40	− 3.54	− 10.51; 3.43	0.31
T/T	2.40	− 8.29; 13.08	0.66	− 0.64	− 8.30; 7.02	0.87	− 2.12	− 11.66; 7.42	0.66
rs368234815									
T/ΔG	0.18	− 7.77; 8.13	0.97	1.16	− 4.48; 6.81	0.68	− 1.58	− 8.62; 5.45	0.66
ΔG/ΔG	0.61	− 8.35; 9.57	0.89	3.42	− 2.94; 9.77	0.29	− 4.24	− 12.16; 3.69	0.29
rs12979860									
C/T	3.19	− 2.94; 9.31	0.30	0.45	− 4.73; 5.64	0.86	− 7.59	− 15.16; − 0.03	0.049
T/T	6.78	− 1.60; 15.16	0.11	− 3.96	− 11.05; 3.14	0.27	− 5.82	− 16.18; 4.53	0.27
rs368234815									
T/ΔG	2.15	− 3.96; 8.27	0.48	0.86	− 4.44; 6.17	0.75	− 3.19	− 10.89; 4.50	0.41
ΔG/ΔG	7.17	0.28; 14.06	0.042	− 0.53	− 6.50; 5.44	0.86	− 7.62	− 16.29; 1.05	0.08

C/C is the reference group for rs12979860 and T/T is the reference group for rs368234815

## Discussion

This is the first study to describe associations between *IFNL* polymorphisms and immunological outcomes in ART-treated PLWH, individuals with undetectable viral loads on ART and homozygous for rs368234815 [ΔG/ΔG] displayed an increased likelihood of normalisation of the CD4^+^:CD8^+^ T-cell ratio, as well as higher proportions of naïve CD8^+^ T-cells and lower CD4^+^ effector memory cells in those with HIV mono-infection. Taken together with previous literature [9, 30], these data are consistent with an overall more favourable immunological profile in the setting of long-term treated HIV-1 infection in those with the rs368234815 [ΔG/ΔG] genotype; the presence of which is linked with both enhanced IFNL4 activity and Th1 responses [31–33].

Our findings are consistent with recent reports of more frequent rs368234815 [ΔG/ΔG] genotypes (IFNL4 expressing) among PLWH classified as long-term non-progressors [34]. While the authors of this report hypothesized that increased T-cell activation and IFN-mediated thymic dysfunction among those with the rs368234815 [TT/TT] genotype, known to be associated with higher interferon-stimulated gene expression (ISG) [20], may contribute to CD4^+^ T-cell decline and a loss of host immune control of HIV-1 infection, it is also possible that these observations relate to better CD8^+^ mediated T-cell control of viral infection in those with ΔG/ΔG who express IFNL4, a view that would be supported by our study results.

Naïve CD8^+^ T-cell subsets were significantly increased in those with HIV mono-infection who carried the rs368234815 ΔG/ΔG homozygous genotype. Previous data has established roles for naïve CD8^+^ T-cells in controlling immune responses to infection. In animal models, bacterial sepsis leads to a rapid depletion of naïve CD8^+^ T-cells, which persists long after resolution of the infection [35]. Previously, we, and others, have reported associations between a higher percentage of naïve CD8^+^ T-cells and higher CD4^+^:CD8^+^ T-cell ratio in long-term treated HIV-1 infection, consistent with better immune recovery with ART [9, 10].

A deeper understanding of how naïve CD8^+^ T-cells influence immune responses arising after viral infection is critical for the development of enhanced therapeutic and vaccination strategies to exploit CD8^+^ T-cell-mediated immunity [36], such as those proposed in HIV-cure strategies. Vaccine immunogens based on conserved HIV epitopes aim to induce CD8^+^ cytotoxic T lymphocytes to kill virus-infected cells and are a target for therapeutic strategies aimed at eradication of HIV reservoirs. Our data suggest that host immunogenetics at the *IFNL* locus may influence CD8^+^ T-cell responses in those with long-term treated HIV-1 infection, with those with rs368234815 [ΔG/ΔG] homozygosity having more favourable immune responses on ART. Given the limited success of HIV vaccine research [37], our data would suggest that host profiling based on IFNL genotype may identify those individuals who may respond more favorably to immune-based therapies, although this hypothesis would require further exploration in prospective studies.

Our study has limitations. The single centre, cross-sectional nature of the study design cannot determine causality, with mortality patterns and other unmeasured factors potentially introducing biases and some ethnicities underrepresented. Our findings also require validation in other more diverse cohorts, testing validity within different ethnicities and treatment environments. In addition, although we measured immune markers that have been associated with more favorable clinical outcomes, we did not include more detailed functional markers of immune function such as measurements of cytokines, ISGs and or IFN classes. Lastly, our sample size limited the ability to explore the impact of different ART regimes on observed relationships. Nevertheless, our novel findings provide further insights consistent with previous studies demonstrating an important role for IFNL4 in the control of chronic HIV-1 infection and further build on emerging data on the importance of CD8^+^ T-cell responses and the CD4^+^:CD8^+^ T-cell ratio in determining response to long-term treated HIV-1 infection.

## Conclusion

In summary, in virally-suppressed, long-term ART-treated PLWH, rs368234815 ΔG/ΔG homozygotes were more likely to have attained normalisation of their CD4^+^:CD8^+^ T-cell ratio, displayed lower CD4^+^ effector memory and higher naive CD8^+^ T-cells. Further studies are needed to replicate our findings in other, larger and more diverse cohorts and to determine the impact of *IFNL* genetic variation on CD4^+^:CD8^+^ T-cell ratio normalisation and clinical outcomes in people living with HIV.

## Supplementary information

**Additional file 1: Figure S1.** Gating strategy used to discriminate CD4^+^ and CD8^+^ T-cell subsets. Note: In the dot Plot of CD62L PE versus CD45RO APC, Q1 displays CD62L^+^CD45RO^−^cells (naïve cells). Q2 displays CD62L^+^ CD45RO^+^ cells (central memory cells), Q3 displays CD62L^−^CD45RO^−^ cells (revertant memory cells), Q4 displays CD62L^−^CD45RO^+^ cells (effector memory cells).

## Data Availability

The datasets generated and/or analysed during the current study are not publicly available due to ethical approval restrictions, but are available from the corresponding author on reasonable request.

## Abbreviations

AIDS: Acquired immunodeficiency syndrome
ART: Antiretroviral therapy
CMV: Cytomegalovirus
DNA: Deoxyribonucleic acid
EDTA: Ethylenediaminetetraacetic acid
HCV: Hepatitis C virus
HIV: Human immunodeficiency viruses
HLA: Human leukocyte antigen
IDU: Injecting drug-users
IFNL: Interferon lambda
ISG: Interferon-stimulated gene expression
IL: Interleukin
InSTI: Integrase strand transfer inhibitors
IQR: Interquartile range
MSM: Men who have sex with men
OR: Odds ratio
PCR: Polymerase chain reaction
PEG/RBV: Pegylated interferon-alpha/ribavirin
PLWH: People living with HIV
RNA: Ribonucleic acid
SNP: Single nucleotide polymorphism
TSLP: Thymic stromal lymphopoietin
