# Supplementary figures and images for: Interferon lambda rs368234815 ΔG/ΔG is associated with higher CD4+:CD8+ T-cell ratio in treated HIV-1 infection
Source: AIDS Res Ther. 2020 Apr 15;17:13. doi: 10.1186/s12981-020-00269-0 (PMC7194102; doi:10.1186/s12981-020-00269-0)

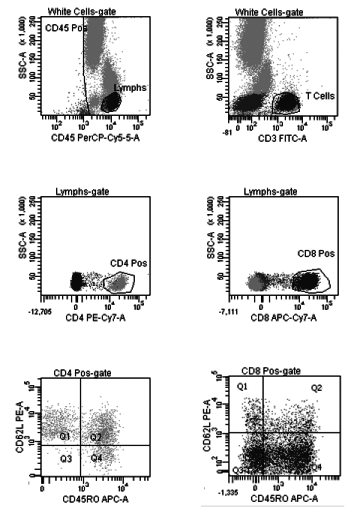

Supplement: Supplementary file 1 — Additional file 1: Figure S1. Gating strategy used to discriminate CD4+ and CD8+ T-cell subsets. Note: In the dot Plot of CD62L PE versus CD45RO APC, Q1 displays CD62L+CD45RO−cells (naïve cells). Q2 displays CD62L+ CD45RO+ cells (central memory cells), Q3 displays CD62L−CD45RO− cells (revertant memory cells), Q4 displays CD62L−CD45RO+ cells (effector memory cells). [file 12981_2020_269_MOESM1_ESM.tif]
